# Three-dimensional evaluation of root dimensions and alveolar ridge width of maxillary lateral incisors in patients with unilateral agenesis

**DOI:** 10.1186/s40510-016-0144-y

**Published:** 2016-10-10

**Authors:** Sharifah AlRushaid, Taranpreet Chandhoke, Achint Utreja, Aditya Tadinada, Veerasathpurush Allareddy, Flavio Uribe

**Affiliations:** 1 Private practice Kuwait City, Kuwait; former resident Division of Orthodontics,Department of Craniofacial Sciences, University of Connecticut School of Dental Medicine, Farmington, Connecticut USA; 2 Division of Orthodontics, Department of Craniofacial Sciences, University of Connecitcut School of Dental Medicine, Farmington, Connecticut USA; 3 Department of Orthodontics, Indiana University, Indianapolis, Indiana USA; 4 Division of Oral and Maxillofacial Radiology, Department of Oral Health and Diagnostic Sciences, University of Connecticut School of Dental Medicine, Farmington, Connecticut USA; 5 College of Dentistry, Department of Orthodontics, The University of Iowa, Iowa City, Iowa USA

## Abstract

**Background:**

The objective of this retrospective case-control study was to measure the maxillary lateral incisor root dimensions and quantify the labial and palatal bone in patients with unilateral maxillary lateral incisor agenesis (MLIA) after orthodontic treatment and compare them to non-agenesis controls using cone beam computed tomography.

**Methods:**

The labiopalatal and mesiodistal root dimensions, mesiodistal coronal dimensions, and labiopalatal bone and alveolar ridge widths of the maxillary lateral incisor were assessed on posttreatment cone beam computed tomography scans of 15 patients (mean age 16.5 ± 3.4 years, 9 females and 6 males) with maxillary lateral incisor agenesis and 15 gender-matched patients (mean age 16.08 ± 3.23 years) with no dental agenesis or anterior Bolton discrepancy. The Mann-Whitney test was used to distinguish any differences in root width, crown width, or changes in labial or palatal bone width between the two groups.

**Results:**

The median labiopalatal root width was narrower in the MLIA group at the level of the cementoenamel junction (CEJ) to 8 mm apical of the CEJ compared to controls (*p* ≤ 0.009). The mesiodistal root width was significantly reduced in the MLIA group at the CEJ and at 4 mm apical to the CEJ. The labiopalatal alveolar ridge width was significantly decreased at 2 mm apical to the CEJ in MLIA group. The mesiodistal crown width was significantly smaller in the MLIA group at both the incisal edge and at the crown midpoint. The bone thickness was similar in both groups.

**Conclusions:**

Coronal and root dimensions in patients with MLIA were reduced compared to controls. Alveolar ridge width was also reduced in patients with MLIA, although bone thickness was not different than controls.

## Background

Tooth *agenesis*, defined as the congenital absence of one or more teeth, is the most common developmental anomaly [1, 2]. Maxillary lateral incisor agenesis (MLIA) is one of the most common forms of dental agenesis. With the exception of the third molars, the maxillary lateral incisor is the second most affected tooth, with the mandibular second premolar agenesis slightly more common [1, 3, 4]. The prevalence of MLIA in the permanent dentition ranges from 1 to 4 % [4] depending on gender, race, and continent.

The agenesis of the maxillary lateral incisor is often associated with other forms of dental anomalies such as microdontia of the contralateral incisor [5–8]. The reduction in tooth size can be noted in both the buccolingual and mesiodistal dimensions, but is typically more prominent in the buccolingual dimension [9]. A recent study found that patients with MLIA had smaller teeth overall compared to controls, with the exception of the maxillary first molars. The average difference in the mesiodistal width of the maxillary and mandibular central incisors in the patients with MLIA was 0.47 and 0.43 mm, respectively [10]. In another study, the maxillary anterior teeth were found to be 0.33 mm smaller in the MLIA patients compared to controls [11]. It should be noted that both of these studies assessed the mesiodistal dimension of the crown using dental casts, which only allows assessment of crown dimensions and not the entire tooth including the root. Three-dimensional imaging could provide additional data on the crown dimensions from multiple planes of space as well as information on the root dimensions in cases with dental anomalies. This has been shown with a recent cone beam computed tomography (CBCT) study comparing subjects with palatally displaced canines (PDCs) to controls which illustrated that the maxillary lateral incisor crown as well as root width was significantly reduced in the buccolingual dimension in patients with PDCs [12].

With most of the current literature in MLIA patients focusing on dental cast measurements and assessing predominantly the differences in crown morphology, there is a need to examine root morphology and changes to the alveolar bone as a result of MLIA. Therefore, the objective of this study was to evaluate the dimensions of the existing maxillary lateral incisor crown and root in patients with unilateral MLIA. In addition, our objectives quantify the amount of the labial and palatal bone in relation to the present lateral incisor in subjects with unilateral MLIA and non-agenesis controls using CBCT.

## Methods

This study was a case-control retrospective evaluation of patient records. Institutional review board approval was granted by the University of Connecticut (IRB 14-015-2) prior to the start of the study. CBCTs were obtained from three private orthodontic offices (Dr. Sheeba Zaidi in Wallingford, CT; and Dr. Carl Roy’s offices in Virginia Beach and Chesapeake, VA) and one periodontic office (Dr. Scott Ross in Miami, FL).

The inclusion criteria for the study group were (1) unilateral maxillary lateral incisor agenesis (MLIA), (2) ≥10 years old at the time of initial records with completed maxillary lateral incisor root formation, (3) no systemic or health problems, and (4) availability of posttreatment CBCT scans of good quality. The exclusion criteria for the study group included (1) bilateral MLIA, (2) history of trauma, (3) root canal therapy, restorations, or incisal edge abrasion of the maxillary lateral incisor, (4) previous root resorption, and (5) patients with cleft palate or any other dentofacial deformities.

Inclusion criteria for the control group were (1) complete eruption of maxillary lateral incisors with complete root formation, (2) absence of abnormal morphology or reduced size of lateral incisors or other teeth, (3) no systemic health problems, and (4) a posttreatment CBCT scans of good quality. The exclusion criteria for the control group were (1) history of trauma, (2) root canal therapy, restorations or incisal edge abrasion of the maxillary incisors, (3) dental agenesis, or (4) Bolton index >1 SD based on the widths of the six anteriors (77.2 ± 2.8).

Approximately, 7000 patient records (clinical examination notes, dental radiographs, photographs, and CBCT scans) were searched of which 56 subjects were identified with unilateral MLIA. Of these 56 subjects, 37 subjects were excluded because of CBCT images were unavailable, two subjects were excluded due to severe root resorption on the maxillary lateral incisor, one subject had congenitally missing lower incisors, and one subject had poor-quality posttreatment CBCT. The study group therefore comprised 15 subjects (9 females and 6 males), with an average age of 16.5 ± 3.4 years (range 12–27 years) at the end of treatment. These subjects were gender matched with 15 subjects (9 females and 6 males) with no MLIA or Bolton discrepancy, which had received orthodontic treatment and served as controls. The average age of the control group was 16.08 ± 3.23 years (range 12.4–25.8) at the end of treatment. A summary of patients’ descriptive characteristics can be found in Table 1.

**Table 1:** Descriptive patient characteristics for study and control group

		Study group	Control group
		Frequency (%)	Frequency (%)
		15	15
Sex	Female	9 (60)	9 (60)
	Male	6 (40)	6 (40)
Age	10–12 yr	1 (6.7)	0
	12.1–14 yr	0	2 (13.3)
	14.1–16 yr	5 (33.3)	7 (46.6)
	16.1–30 yr	9 (60)	6 (40)
Side of agenesis	R	7 (46.6)	0
	L	8 (53.3)	0
Treatment of agenesis	Space opening	12 (80)	0
	Space closure	3 (20)	0

*Yr* years, *R* right, *L* left

Twenty-nine CBCT images were obtained from subjects using the Classic i-CAT (14-bit gray-scale resolution, 0.3 mm voxel size), cone beam 3-D dental imaging system and reconstructed through i-CAT Vision software (Imaging Sciences International, Hatfield, PA). One CBCT was produced with Picasso-Trio (14-bit gray-scale resolution, 0.2 mm voxel size) cone beam 3-D dental imaging system and reconstructed through Ez3D Plus software (VATECH Global, Korea). All images were transported as digital imaging and communications in medicine (DICOM) files and imported into Dolphin Imaging software 3D (version 11.0; Dolphin Imaging and Management Solutions, Chatsworth, CA) for secondary reconstruction.

All measurements were made on the maxillary lateral incisor on the non-agenesis side for all subjects in the MLIA group. For the control group, the measurements were done on both the right and left maxillary lateral incisors and the mean value was recorded. Measurements were made on the multiplanar view. All sagittal, axial, and coronal CBCT sections were analyzed with slice thickness of 1 voxel and measured to the nearest 0.1 mm.

**Fig. 1:**
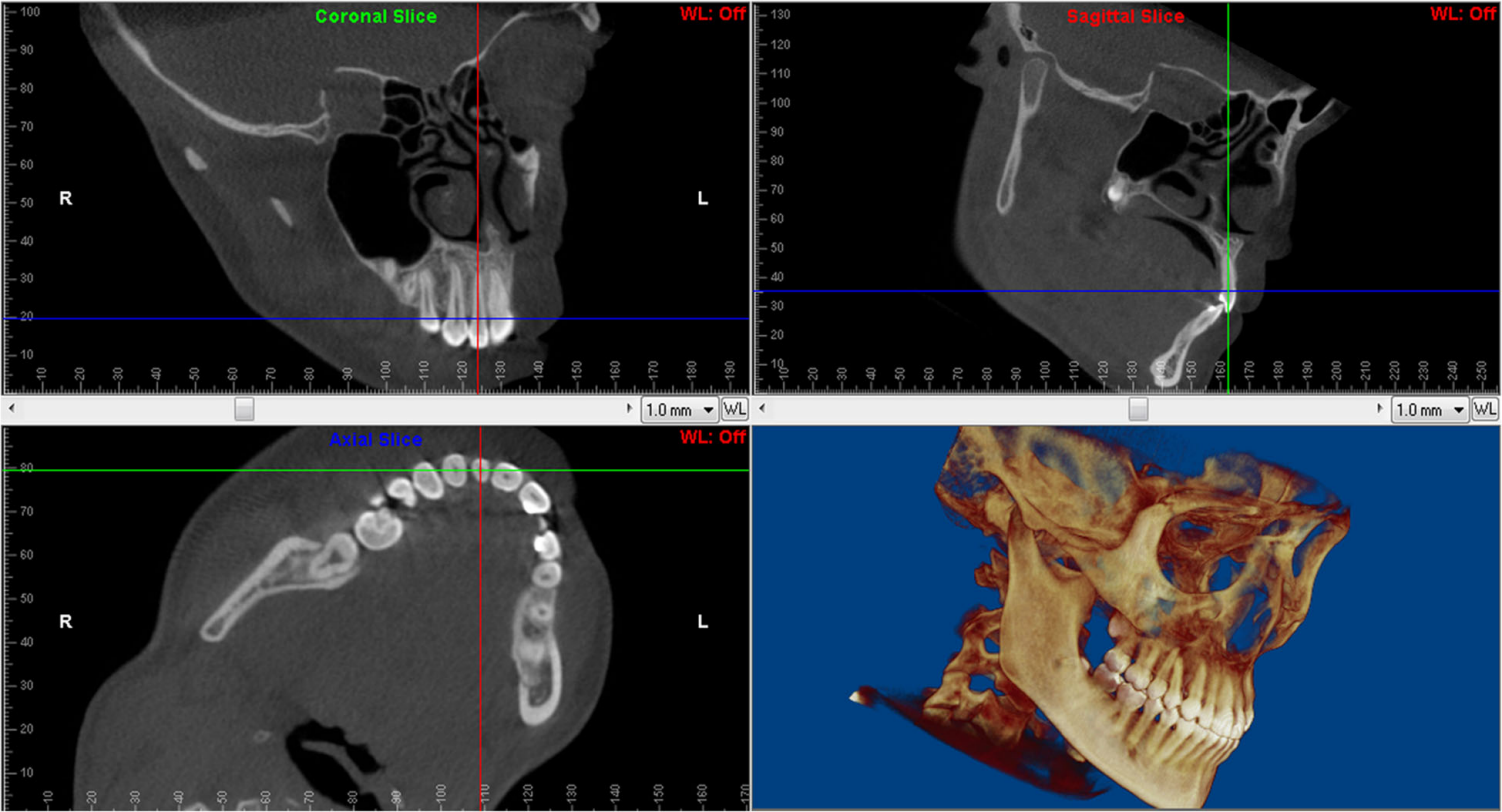
Volumetric rendering, sagittal, coronal, and axial sections parallel to the long axis of maxillary central incisor, through the center of the incisor

The multiplanar view and the volumetric rendering were used to identify the long axis and the center of the incisor root (Fig. 1). Images were reoriented so that the lateral incisor was positioned vertically with the root canal parallel to the software’s vertical line in both sagittal and coronal slices.

### Variables measured

**Fig. 2:**
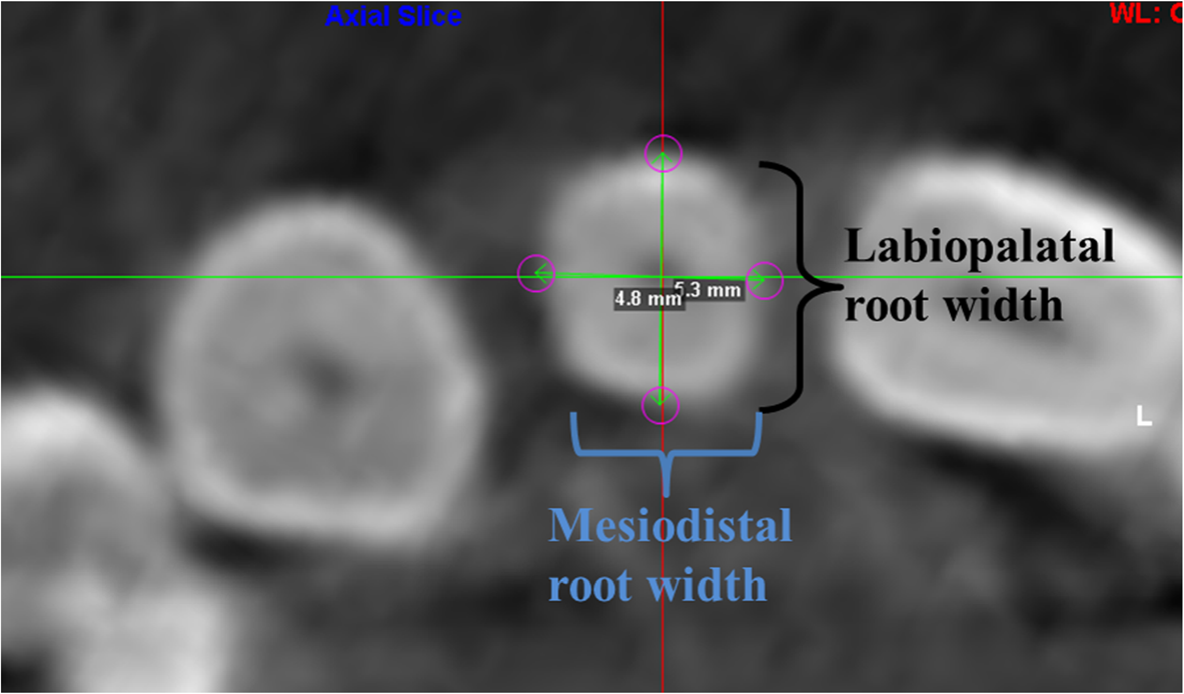
Labiopalatal (LP) and mesiodistal (MD) root widths of the lateral incisor at level of cementoenamel junction (CEJ)

Two methods were used to assess the labiopalatal and mesiodistal root width. In the first method, the measurements were made using axial sections. The lateral incisor root width was measured using the method described by Liuk et al. [12]. Measurements were made on three axial sections taken perpendicular to the long axis of the tooth as determined by the sagittal section: at the cementoenamel junction (CEJ), 4 mm apical to CEJ, and 8 mm apical to CEJ of the maxillary lateral incisor. The labiopalatal root thickness was measured on axial slices across the root from the labial-most surface of the incisor root to the palatal-most surface of the incisor root. The mesiodistal root width was measured from the widest point on the mesial surface to the widest point on the distal surface (Fig. 2).

**Fig. 3:**
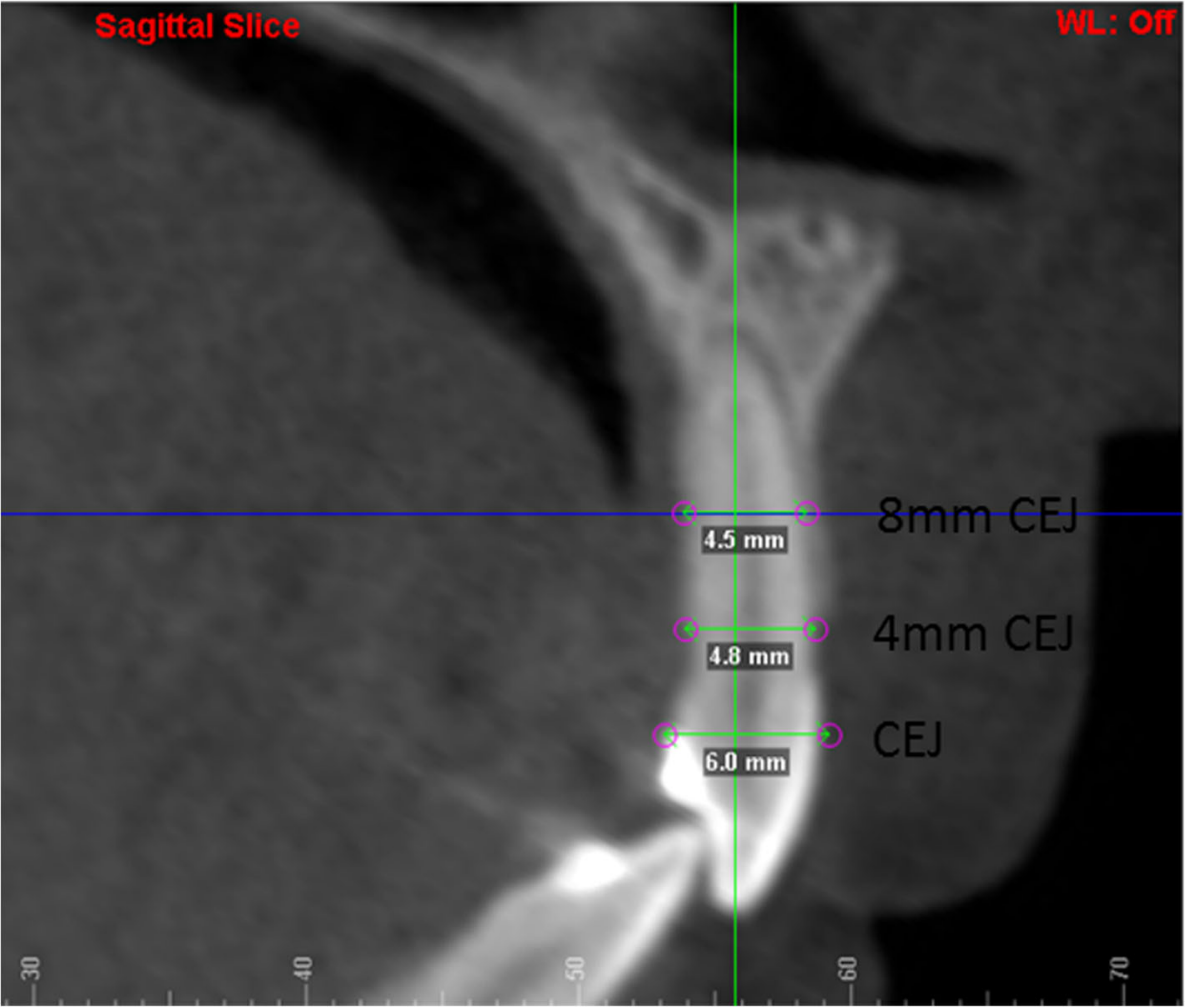
Labiopalatal root width on sagittal section at the CEJ, 4 mm apical to CEJ and 8 mm apical to CEJ

**Fig. 4:**
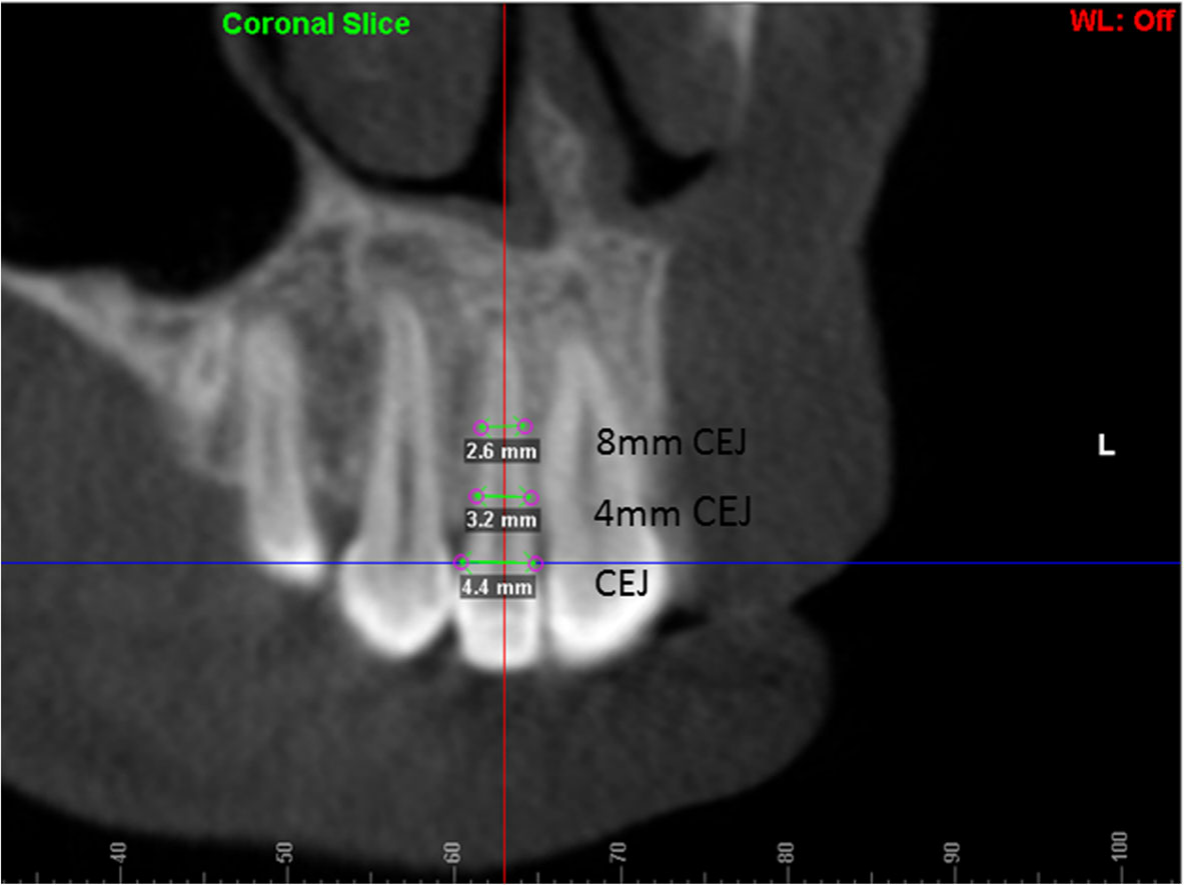
Mesiodistal root width on coronal section at the CEJ, 4 mm apical to CEJ and 8 mm apical to CEJ

In the second method, the measurements were made using sagittal sections. The labiopalatal root width was measured on the sagittal section parallel to the long axis of the lateral incisor through the center of the root. These measurements were again at the CEJ, 4 mm apical to the CEJ, and 8 mm apical to the CEJ of the lateral incisor. Root width was measured from the labial to the palatal root surface of the lateral incisor (Fig. 3). For the mesiodistal root width, the coronal section was utilized with the section made parallel to the long axis of the lateral incisor, through the center of the root. Measurements were done at the same three levels: at the CEJ, and 4 and 8 mm apical to the CEJ. Root width was measured from distal-most to mesial-most incisor root surface (Fig. 4).

**Fig. 5:**
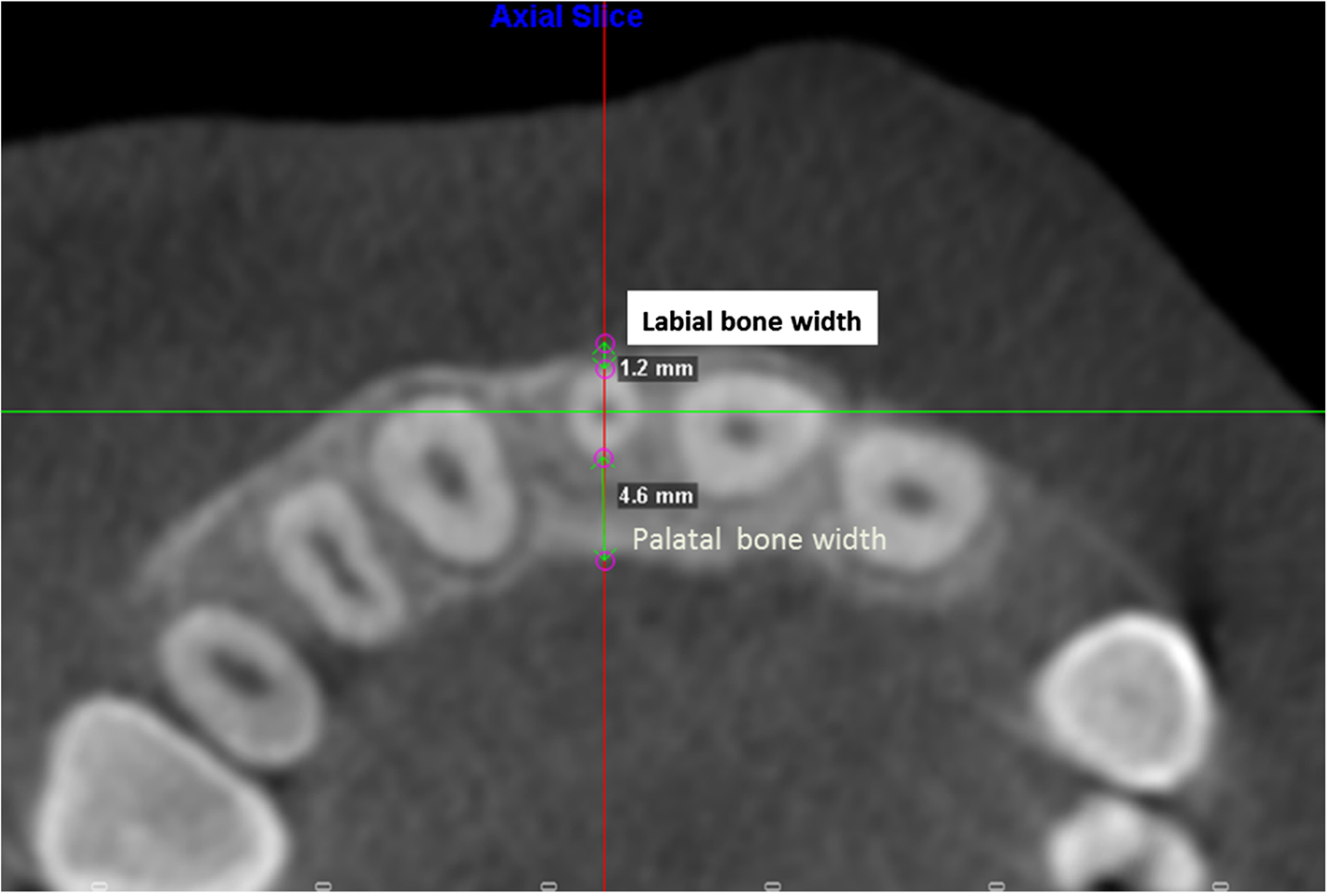
Labial and palatal bone width on axial slice 6 mm apical to CEJ

The labiopalatal thickness of the maxillary lateral incisor alveolar bone was measured using two methods. In the first method, again approached from the axial perspective, the measurements were taken with four axial sections made perpendicular to the long axis of the lateral incisor (Fig. 5. Labial and palatal bone width on axial slice 6 mm apical to CEJ). Four measurements were taken on the labial surface and four on the palatal surface of the lateral incisor. The first axial section was made 2 mm apical to the CEJ as determined by the sagittal section of the lateral incisor. The second axial section was taken 4 mm apical to the CEJ; the third axial section was taken 6 mm apical to the CEJ of the tooth; and the fourth axial section at 10 mm apical to the CEJ of the tooth. The labial bone thickness was determined by a line from the labial-most limit of the buccal bone to the outermost labial surface of the incisor. The palatal bone thickness was measured by a line from the palatal-most limit of the bone to the outermost palatal surface of the incisor.

In the second method, the labiopalatal alveolar bone was measured on the sagittal section parallel to the long axis of the lateral incisor root. Measurements were obtained again at 2, 4, 6, and 10 mm apical to the CEJ.

The total labiopalatal bone width was determined mathematically by adding labial bone width to the palatal bone width as determined by the axial slices on the scans. The labiopalatal ridge width was measured on the sagittal section at 2, 4, 6, and 10 mm apical to the CEJ. The ridge width was measured from the labial-most limit of the labial bone to the palatal-most limit of the palatal bone. These landmarks were verified on axial slices at each of the four levels.

**Fig. 6:**
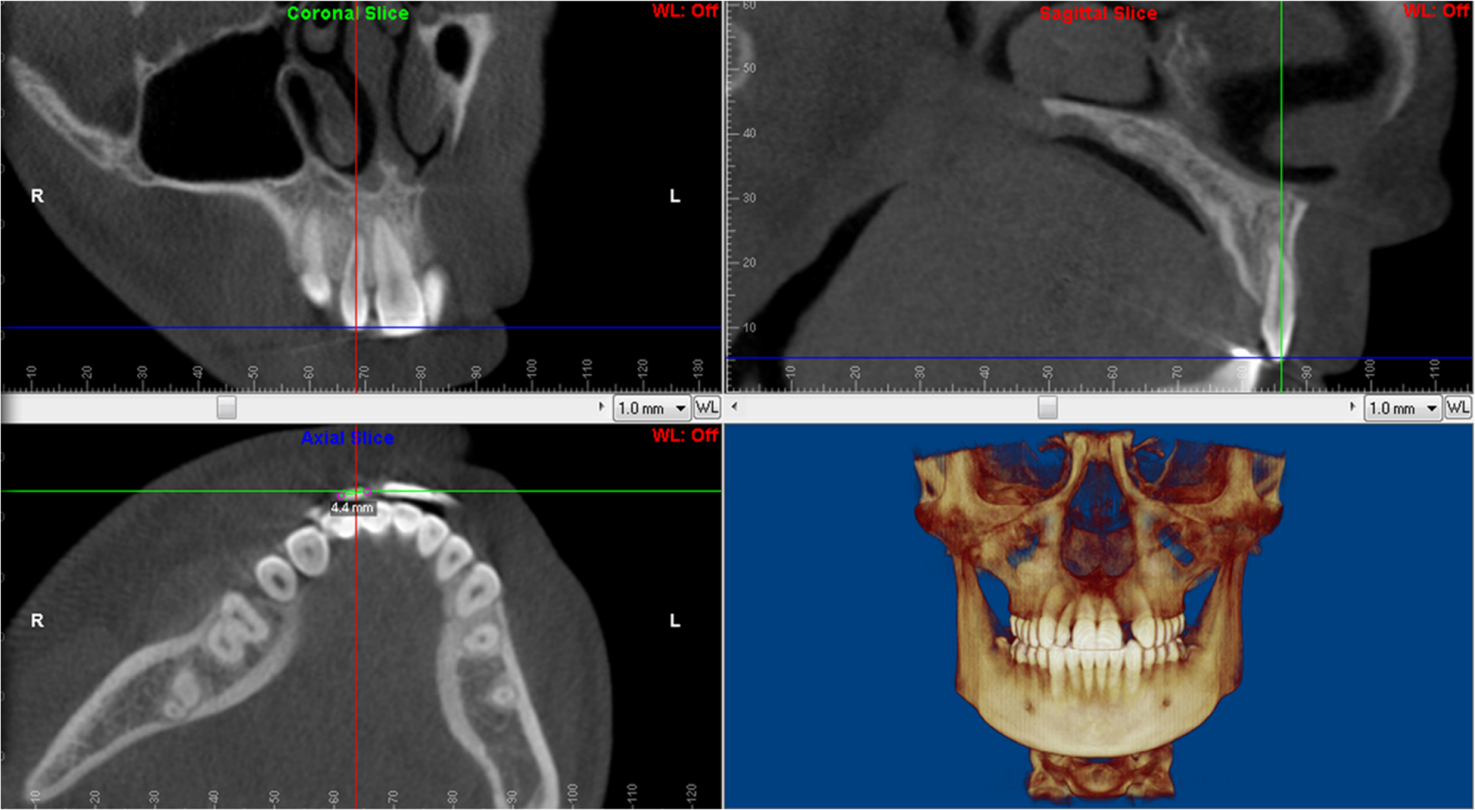
The multiplanar, sagittal, coronal and axial views parallel to long axis of the tooth used to measure the incisal edge on axial sections

The mesiodistal crown width was measured on two axial sections made perpendicular to the long axis of the lateral incisor. The first axial section was made at the level of incisal edge as determined by the sagittal section (Fig. 6). The second axial section was made at the crown’s midpoint between the incisal edge and the CEJ. The mesiodistal crown width was measured by a method similar to that described by Benninger et al., from the widest identifiable point on the mesial surface to the widest identifiable point on the distal surface of the incisor crown [13].

### Statistical analysis

Simple descriptive statistics were used to summarize and present the data. The reliability of measurements was computed for treatment and control groups separately by using Cronbach’s alpha (intra-class correlation coefficients). Cronbach alpha values were computed for each outcome variable. Data distribution was assessed by the Kolmogorov-Smirnov test for normality. Since the data were skewed, non-parametric tests (Mann-Whitney *U* tests) were used to examine the distribution in outcomes between the treatment and control groups. All tests were two-sided. Since multiple outcomes were assessed, Bonferroni corrections were used to minimize type 1 errors. Depending on the number of outcomes assessed, the *p* value that deemed to be statistically significant was set. For example, when three outcomes were assessed, a *p* value of <0.017 was deemed to be statistically significant and when four outcomes were assessed, a *p* value of <0.012 was deemed to be statistically significant. All statistical analyses were computed using SPSS Version 22.0 software (IBM Corp, New York, NY).

## Results

The summary of Cronbach alpha estimates examining the internal consistency (for both MLIA and control groups) of all the outcome measures are presented in Table 2. Overall, all of the measures had high reliability. Cronbach alpha values could not be computed for two variables in the MLIA group (labial bone, 2 mm apical to CEJ [axial method] and labial bone, 2 mm apical to CEJ [sagittal method]) since the items had zero variance between the two sets of measurements.

**Table 2:** Reliability analysis for the different measurements: Cronbach alpha values

Variable	Treatment group	Control group
LP At CEJ, method 1 (axial)	0.982	0.949
LP 4 mm apical to CEJ, method 1 (axial)	0.976	0.919
LP 8 mm apical to CEJ, method 1 (axial)	0.947	0.959
MD At CEJ, method 1 (axial)	0.850	0.901
MD 4 mm apical to CEJ, method 1 (axial)	0.872	0.924
MD 8 mm apical to CEJ, method 1 (axial)	0.962	0.966
LP At CEJ, sagittal	0.989	0.932
LP 4 mm apical to CEJ, sagittal	0.985	0.943
LP 8 mm apical to CEJ, sagittal	0.971	0.938
MD at CEJ, coronal	0.809	0.831
MD 4 mm apical to CEJ, coronal	0.958	0.912
MD 8 mm apical to CEJ, coronal	0.969	0.962
Labial bone 2 mm apical to CEJ, method 1 (axial)	Scale has zero variance items	0.756
Labial bone 4 mm apical to CEJ, method 1 (axial)	0.739	0.936
Labial bone 6 mm apical to CEJ, method 1 (axial)	0.921	0.876
Labial bone 10 mm apical to CEJ, method 1 (axial)	0.943	0.964
Palatal bone 2 mm apical to CEJ, method 1 (axial)	0.995	0.829
Palatal bone 4 mm apical to CEJ, method 1 (axial)	0.900	0.895
Palatal bone 6 mm apical to CEJ, method 1 (axial)	0.956	0.991
Palatal bone 10 mm apical to CEJ, method 1 (axial)	0.981	0.989
Labial bone 2 mm apical to CEJ, method 2 (sagittal)	Scale has zero variance items	0.997
Labial bone 4 mm apical to CEJ, method 2 (sagittal)	0.981	0.936
Labial bone 6 mm apical to CEJ, method 2 (sagittal)	0.964	0.974
Labial bone 10 mm apical to CEJ, method 2 (sagittal)	0.978	0.992
Palatal bone 2 mm apical to CEJ, method 2 (sagittal)	0.998	0.971
Palatal bone 4 mm apical to CEJ, method 2 (sagittal)	0.987	0.968
Palatal bone 6 mm apical to CEJ, method 2 (sagittal)	0.990	0.981
Palatal bone 10 mm apical to CEJ, method 2 (sagittal)	0.987	0.997
LP ridge width 2 mm apical to CEJ	0.997	0.984
LP ridge width 4 mm apical to CEJ	0.998	0.940
LP ridge width 6 mm apical to CEJ	0.995	0.964
LP ridge width 10 mm apical to CEJ	0.996	0.986
MD crown width at IE	0.937	0.795
MD crown width at midpoint	0.897	0.894

*LP* labiopalatal, *MD* mesiodistal, *CEJ* cementoenamel junction. The scale had zero variance and therefore was unable to determine ICC as all values were zero

The results of the Mann-Whitney *U* tests examining the distribution of outcomes between the MLIA and control groups are summarized in Tables 3, 4, and 5. Fifteen outcome measures were distributed significantly different between the MLIA and control groups. When assessing labiopalatal root width by the axial method, those in the MLIA group had lower median values than those in the control group at the CEJ, 4 mm apical to CEJ, and 8 mm apical to CEJ (*p* ≤ 0.009). Similarly, those in the MLIA group had lower median values for the mesiodistal root width at CEJ and 4 mm apical to CEJ (*p* ≤ 0.003) by the axial method. With the sagittal method, those in the MLIA group had lower values for labiopalatal root width at CEJ, 4 mm apical to CEJ, mesiodistal root width at CEJ, and mesiodistal root width 4 mm apical to CEJ when compared to those in the control group (*p* ≤ 0.003). Those in the MLIA group had lower coronal width values at IE and at the midpoint when compared to those in the control groups (*p* ≤ 0.0001), Table 3.

**Table 3:** Lateral incisor measurements from axial and sagittal sections

	Group	Mean	SD	25th percentile	50th percentile	75th percentile	*p*
Axial method							
Labiopalatal root width (mm)							
At CEJ	MLIA	5.86	0.71	5.60	5.90	6.10	<0.0001*
	Control	7.24	0.43	6.90	7.20	7.65	
4 mm apical to CEJ	MLIA	4.99	0.64	4.60	4.99	5.50	<0.0001*
	Control	5.97	0.43	5.50	6.05	6.25	
8 mm apical to CEJ	MLIA	4.06	0.69	3.50	4.00	4.60	0.009*
	Control	4.86	0.65	4.30	4.65	5.50	
Mesiodistal root width (mm)							
At CEJ	MLIA	4.91	0.31	4.70	4.90	5.20	<0.0001*
	Control	5.66	0.54	5.30	5.55	6.15	
4 mm apical to CEJ	MLIA	3.86	0.42	3.50	3.80	4.30	0.003*
	Control	4.57	0.69	4.05	4.55	4.95	
8 mm apical to CEJ	MLIA	3.15	0.42	2.80	3.00	3.30	0.092
	Control	3.57	0.68	2.95	3.30	4.20	
Sagittal method							
Labiopalatal root width (mm)							
At CEJ	MLIA	5.94	0.72	5.60	5.90	6.30	<0.0001*
	Control	7.21	0.40	6.90	7.20	7.55	
4 mm apical to CEJ	MLIA	5.01	0.63	4.60	5.00	5.60	<0.0001*
	Control	5.94	0.42	5.55	5.85	6.35	
8 mm apical to CEJ	MLIA	4.12	0.70	3.50	4.30	4.60	0.018
	Control	4.87	0.67	4.45	4.80	5.60	
Mesiodistal root width (mm)							
At CEJ	MLIA	4.85	0.27	4.60	4.90	5.00	<0.0001*
	Control	5.74	0.52	5.35	5.70	6.20	
4 mm apical to CEJ	MLIA	3.83	0.43	3.50	3.60	4.20	0.003*
	Control	4.53	0.67	3.95	4.50	5.00	
8 mm apical to CEJ	MLIA	3.06	0.43	2.70	3.00	3.30	0.05
	Control	3.53	0.67	2.90	3.30	4.10	
Width at incisal edge (IE, mm)	MLIA	4.40	0.82	3.90	4.40	4.90	<0.0001*
	Control	6.16	0.51	5.90	6.00	6.60	
Width at midpoint (mm)	MLIA	5.45	0.95	5.20	5.60	5.90	<0.0001*
	Control	7.00	0.23	6.75	7.05	7.20	

Since three distance points from CEJ were used to examine the outcomes, in order to minimize type 1 errors arising from multiple outcome assessments (at CEJ, 4 mm apical to CEJ, and 8 mm apical to CEJ), adjustments (based on Bonferroni formula) were made to *p* values to be deemed statistically significant. A *p* value of <0.017 was deemed to be statistically significant

*p value is statistically significant at *p* < 0.017

**Table 4:** Bone thickness parameters measured from sagittal sections

	Group	Mean	SD	25th percentile	50th percentile	75th percentile	*p*
Sagittal method							
Labial bone thickness (mm)							
2 mm apical to CEJ	MLIA	0.00	0.00	0.00	0.00	0.00	0.317
	Control	0.10	0.40	0.00	0.00	0.00	
4 mm apical to CEJ	MLIA	0.52	0.57	0.00	0.40	1.00	0.015
	Control	1.04	0.48	0.65	1.05	1.15	
6 mm apical to CEJ	MLIA	1.24	0.55	1.00	1.20	1.50	0.983
	Control	1.27	0.68	0.75	1.35	1.40	
10 mm apical to CEJ	MLIA	1.61	0.65	1.20	1.30	1.90	0.328
	Control	1.81	0.92	1.25	1.80	2.10	
Palatal bone thickness (mm)							
2 mm apical to CEJ	MLIA	0.13	0.35	0.00	0.00	0.00	0.229
	Control	0.27	0.44	0.00	0.00	0.00	
4 mm apical to CEJ	MLIA	1.03	0.94	0.00	0.90	1.50	0.771
	Control	1.09	0.73	0.50	1.15	1.45	
6 mm apical to CEJ	MLIA	1.83	1.23	1.00	1.20	2.20	0.740
	Control	1.55	1.03	0.80	1.45	2.15	
10 mm apical to CEJ	MLIA	3.15	1.99	1.60	2.80	4.80	0.575
	Control	2.61	1.82	1.60	1.85	3.75	
Total labiopalatal alveolar ridge width (mm), includes tooth width							
2 mm apical to CEJ	MLIA	5.43	0.99	4.70	5.30	5.80	0.002*
	Control	6.43	0.65	6.00	6.15	6.95	
4 mm apical to CEJ	MLIA	6.43	1.53	5.50	5.90	7.80	0.026
	Control	7.47	0.71	6.80	7.55	7.90	
6 mm apical to CEJ	MLIA	7.33	1.25	6.40	7.40	8.60	0.520
	Control	7.68	1.03	7.00	7.65	8.25	
10 mm apical to CEJ	MLIA	7.90	1.93	6.30	8.50	9.50	0.787
	Control	8.02	1.26	7.35	7.95	8.20	

Since four distance points from CEJ were used to examine outcomes, in order to minimize type 1 errors arising from multiple outcome assessments (at four different levels: 2 mm apical to CEJ, 4 mm apical to CEJ, 6 mm apical to CEJ, and 10 mm apical to CEJ), adjustments (based on Bonferroni formula) were made to *p* values to be deemed statistically significant. A *p* value of <0.012 was deemed to be statistically significant

*p value is statistically significant at *p* < 0.012

**Table 5:** Bone thickness parameters measured from axial sections

	Group	Mean	SD	25th percentile	50th percentile	75th percentile	*p*
Axial method							
Labial bone thickness (mm)							
2 mm apical to CEJ	MLIA	0.00	0.00	0.00	0.00	0.00	0.15
	Control	0.11	0.34	0.00	0.00	0.00	
4 mm apical to CEJ	MLIA	0.41	0.64	0.00	0.00	1.10	0.083
	Control	0.72	0.61	0.35	0.60	1.00	
6 mm apical to CEJ	MLIA	1.13	0.47	0.90	1.20	1.30	0.662
	Control	1.15	0.62	1.00	1.25	1.50	
10 mm apical to CEJ	MLIA	1.53	0.64	1.20	1.20	2.00	0.296
	Control	1.64	0.73	1.25	1.40	1.85	
Palatal bone thickness (mm)							
2 mm apical to CEJ	MLIA	0.13	0.35	0.00	0.00	0.00	0.944
	Control	0.08	0.26	0.00	0.00	0.00	
4 mm apical to CEJ	MLIA	0.77	0.76	0.00	0.60	1.40	0.404
	Control	0.98	0.74	0.45	0.90	1.35	
6 mm apical to CEJ	MLIA	1.64	1.08	0.90	1.10	2.10	0.803
	Control	1.54	0.85	0.95	1.45	1.90	
10 mm apical to CEJ	MLIA	2.83	1.69	1.40	2.60	4.20	0.618
	Control	2.36	1.59	1.35	1.75	3.45	
Total labiopalatal bone thickness (mm)							
2 mm apical to CEJ	MLIA	0.13	0.35	0.00	0.00	0.00	0.631
	Control	0.20	0.46	0.00	0.00	0.00	
4 mm apical to CEJ	MLIA	1.17	1.30	0.00	0.60	2.80	0.190
	Control	1.71	0.75	1.40	1.75	2.20	
6 mm apical to CEJ	MLIA	2.78	1.16	1.80	2.40	3.40	0.771
	Control	2.69	0.91	2.10	2.40	3.00	
10 mm apical to CEJ	MLIA	4.36	1.84	2.50	4.30	6.10	0.693
	Control	4.00	1.46	3.20	3.95	4.40	

Those in the MLIA group had lower values for the labiopalatal ridge at 2 mm apical to CEJ when compared to those in the control group (*p* ≤ 0.002), Table 4.

## Discussion

Several studies have reported the presence of a microdont or a peg-shaped lateral incisor in cases with unilateral MLIA [6, 10, 14]. McKeown et al. [15] found a reduction in crown width in both buccolingual and mesiodistal dimensions in cases with oligodontia. Gungor and Turkkahraman [16] evaluated tooth dimensions in mild and severe hypodontia cases and found that the mesiodistal and buccolingual dimensions of the teeth in both mild and severe hypodontia were smaller. In addition, the maxillary lateral incisor showed the greatest reduction in the mesiodistal dimension. However, all reported studies measured the crown dimension on dental casts using digital calipers and none of the previous studies looked at the root dimension.

This study showed that the labiopalatal root width of the maxillary lateral incisor in the MLIA group was significantly smaller than controls. The labiopalatal root width was on average 1.3 mm smaller than controls at the level of the CEJ, and 0.75 mm at 8 mm apical to the CEJ. The mesiodistal root width was 15 to 12 % smaller in the MLIA group from the CEJ down to 8 mm apical to the CEJ. These findings were consistent with the findings of Liuk et al. [12], who reported that on average, maxillary lateral incisor buccolingual width was 0.7 mm smaller in subjects with palatally displaced canines compared to controls. The authors also reported a smaller reduction of the mesiodistal root width of the maxillary lateral incisor in the palatally displaced canine group.

The crown morphology in the MLIA group varied significantly, where the mesiodistal incisal edge width varied from 2.3 to 5.8 mm with a mean of 4.4 mm. This was significantly lower than the mean mesiodistal width at the incisal edge of 6.2 mm in the control group with a mean difference of 1.8 mm. The mean mesiodistal width at crown midpoint was 5.5 mm in the MLIA group and was 1.5 mm less than the controls. These findings are in support of other studies where microdontia of the maxillary lateral incisor was seen in 40 % of cases with unilateral MLIA [5] and a peg lateral incisor was seen in 20 % of the subjects in a Turkish sample with unilateral MLIA [6]. This is of clinical relevance, as the reduction in clinical crown width should be taken into consideration when planning the best restorative option for the contralateral missing incisor.

The thickness of the labial bone in the esthetic zone is considered the most important factor in determining the best treatment option for MLIA. Adequate facial bone in the anterior maxilla is crucial to create soft tissue profile and prevent future bone resorption when an implant is placed [17, 18]. In our study, the labial and palatal bone thickness was evaluated at four levels: 2 mm apical to the CEJ, 4 mm apical to the CEJ, 6 mm apical to the CEJ, and 10 mm apical to the CEJ on both axial and sagittal slices. Both control and MLIA groups had thin labial bone width at all heights with no significant difference between groups. The mean labial bone width for the MLIA ranged between 0.0 and 1.61 mm and 0.1 and 1.81 mm for the control group. These findings are in accordance with the reported labial bone thickness around healthy maxillary lateral incisors which averaged from 0.5 mm at the level of alveolar crest, to 0.84 mm at 4 mm apical to alveolar crest [18].

The total labiopalatal bone thickness was similar in both groups at 2–10 mm apical to the CEJ. However, the total labiopalatal alveolar ridge width was on average at approximately 1 mm narrower in the MLIA group at 2 and 4 mm apical to the CEJ, and this finding was statistically significant at 2 mm. This may be related to the reduction in the labiopalatal root width of the maxillary lateral incisor in the MLIA group, as the labiopalatal root width was approximately 1 mm narrower at the CEJ and 4 mm apical to the CEJ. This finding is supported by a recent CBCT study in which the alveolar ridge in patients with MLIA was evaluated and no significant difference in ridge width in the edentulous lateral incisor site and ridge width of the contralateral incisor were noted [19]. This suggests that the alveolar ridge width in the edentulous site of patients with MLIA is inherently decreased considering the minimum implant diameter sizes used today in clinical practice. Therefore, adequate ridge dimensions prior to implant placement may always require grafting these sites for adequate esthetics in these patients. Although it has been suggested to graft at the time of implant placement, this bone volume deficiency is unlikely to be restored with this procedure [20]. Placing the implants more palatally may be another option [21]; however, the alveolar ridge labiopalatal width may not allow to achieve 2 mm of labial bone thickness without compromising the implant bone coverage on the palatal aspect. On the other hand, if this labial alveolar width thickness in not achieved, gingival recession with exposure of the labial threads of the implants is likely to occur in the long term [22, 23]. Possibly this alveolar bone width deficiency in patients with thick labial biotype may not be as critical. Alternatively, a soft tissue graft may be considered to increase the soft tissue thickness [24, 25]; however, it may be necessary to obtain adequate labial bone thickness in order to support the adjacent soft tissue [26]. Finally, due to the physical constraints of the area, space closure may be indicated instead of opening in these individuals for a better esthetic result [27]. However, the occlusal relationship, canine’s anatomy, and biomechanical orthodontic considerations are important in this decision-making process.

The use of CBCT is becoming popular in dentistry, particularly in orthodontics as a three-dimensional method for diagnosis and treatment planning. CBCT is considered not only a diagnostic tool but also a measuring instrument in which accuracy is related to smaller voxel size [28]. The validity of CBCT in measuring root width and height has been reported with high degree of accuracy when CBCT root measurements were compared to direct measurements on extracted teeth [13]. Moreover, the accuracy of CBCT crown measurements has been evaluated by Celikoglu et al. [29]. The authors found that the mesiodistal dimensions of anterior teeth and Bolton ratios showed acceptable Pearson’s correlation coefficient when they compared measurements on CBCT and plaster models.

The accuracy of alveolar bone thickness measurements on CBCT has been evaluated by several authors [30–33]. Measurements from the current study were made on the multiplanar view rather than the three-dimensional (3-D) reconstructed image as the virtual renderings are projected images and not actual surfaces [34]. In addition, reliability was found to be higher when landmarks were identified on the multiplanar views compared to the 3-D reconstructed images [35]. Nevertheless, the bone and cementum have similar densities and the accuracy of determining the alveolar bone margin is affected by the physical spatial resolution, the minimum distance needed to distinguish two objects. The spatial resolution for the i-CAT machine was found to be 0.86 mm, meaning the CBCT machine will only be able to detect the difference between two objects if they were 0.86 mm apart [36]. Therefore, the labial bone width in this study may not be fully accurate, specifically at 2 and 4 mm apical to the CEJ.

One of the challenges in this study was the location of the CEJ, which was used for the different reference points. The CEJ of the lateral incisor in this study was identified by the change of tooth outline from the crown to root and change in density and opacity from the enamel to less dense and less opaque cementum [12]. The CEJ on the CBCT is considered an accurate and a reliable landmark [32]. In fact, Leung et al. [32] reported that the CEJ could be identified accurately with a margin of error of at least one voxel size (0.3 mm in our study).

The inclusion of a control group in our study with a similar age and gender distribution is an important factor when drawing conclusions from the results, and thus removing confounding factors that may affect labial and palatal bone width. Nevertheless, sample size and voxel size were the two major shortcomings of this study. To better decide best treatment option for patients with MLIA studies, utilizing higher resolution CBCT with a larger sample size and long-term follow-up are needed.

The present study used a retrospective study design. A true cause-and-effect relationship between the primary independent variables and outcomes cannot be established with a retrospective study design. The data presented in the study was not collected exclusively for the study. Consequently, all confounders could not be accounted for or adjusted in the analyses. Finally, the external validity and generalizability of our study findings is also questionable as the study samples were drawn from only three private practice offices. The study conclusions should be interpreted keeping these inherent limitations in perspective.

## Conclusions

1. The labiopalatal root width of the existing maxillary lateral incisor was significantly smaller in the unilateral MLIA group than in the control group.
2. The mesiodistal root width of the maxillary lateral incisor in the MLIA group was also significantly narrower than controls at the level of the CEJ and 4 mm apical to the CEJ.
3. The labiopalatal alveolar ridge width was significantly narrower (by 15 %) in the MLIA group at 2 mm apical to the CEJ compared to controls.
4. The mesiodistal width of the lateral incisor crown was significantly reduced in the MLIA group when compared to controls.
5. No difference in the total bone thickness was observed between the MLIA group and controls.
